# 

**Published:** 1891-08

**Authors:** 


WHOLE NUMBER, CCCLXI.

New Series.

August, 1891.

VOL. XXXI. No. 1.

LIBRARY Indexed S. G. O.

### BUFFALO MEDICAL AND SURGICAL JOURNAL.

(449) 38

Established 1845 by AUSTIN FLINT, M. D.

PUBLISHED MONTHLY.

**Editors:**

THOS. LOTHROP, M. D. WM. WARREN POTTER, M. D.

**Associate Editors:**

WILLIAM C. KRAUSS, M. D. JAMES WRIGHT PUTNAM, M. D.

JOHN A. MILLER, Ph. D. DeLANCEY ROCHESTER, M. D.

JOHN PARMENTER, M. D.

SURGEON GENL'S OFFICE, LIBRARY.

EUROPEAN AGENCY,

TRÜBNER & CO.,

57 AND 59 LUDGATE HILL, LONDON, E. C.

BUFFALO:
1891.

FOREIGN
SUBSCRIPTION, $2.50

Entered at the Post-Office at Buffalo, N. Y., as Second-class Mail Matter.

*Times Printing House, Buffalo, N. Y.*

Table of Contents on Page V.

